# New metallophamaceutic reduced renal injury induced by non-steroidal anti-inflammatory ^1^

**DOI:** 10.1590/s0102-865020190120000001

**Published:** 2020-02-03

**Authors:** Clóvis Ney Pinheiro Macêdo, Francisco Evanilso Silva Braga, Ana Paula Bomfim Soares Campelo, Gabriel Maia Diniz, Luiz Gonzaga de França Lopes, Marcos Kubrusly, Marcio Wilker Soares Campelo

**Affiliations:** I Fellow Master degree, Postgraduate Program in Minimally Invasive Technology and Health Simulation Area, Medical School, Centro Universitário Christus (UNICHRISTUS), Fortaleza-CE, Brazil. Conception and design of the study, technical procedures, acquisition and interpretation of data, manuscript preparation, critical revision.; II Graduate student, Medical School, UNICHRISTUS, Fortaleza-CE, Brazil. Technical procedures, acquisition of data, manuscript preparation.; III PhD, Department of Surgery, Universidade Federal do Ceará (UFC), Fortaleza-CE, Brazil. Statistical analysis, manuscript preparation, critical revision.; IV PhD, Full Professor, Chemistry Department, UFC, Fortaleza-CE, Brazil. Conception and design of the study, ruthenium synthese, manuscript preparation, critical revision.; V PhD, Full Professor, Medical School, UNICHRISTUS, Fortaleza-CE, Brazil. Conception and design of the study, acquisition and interpretation of data, manuscript preparation, critical revision.

**Keywords:** Anti-Inflammatory Agents, Non-Steroidal, Kidney, Ruthenium, Rats

## Abstract

**Purpose:**

To evaluate the effect of Rut-bpy (Cis-[Ru(bpy)2(SO3)(NO)]PF 6), a novel nitric oxide donor, able to modulate the histological changes caused by the NASID (meloxicam).

**Methods:**

Wistar rats were assigned into three groups (n=6 rats/group): Sham group (saline solution), NSAID group (meloxicam - 15 mg/kg) and Rut-bpy group (100 mg/kg of Rut-bpy associated with 15mg/kg of meloxicam). At the end of experiments, kidneys were removed for histological study, fractal dimension and lacunarity in all animals.

**Results:**

At the histological examination, all animals (six animals – 100 %) in the NSAID group had membrane thickening and other changes (necrosis, acute tubular congestion and vascular congestion); on the other hand, only one animal (16.6 %) of the Rut-bpy group had congestion. The fractal dimension and lacunarity were greater in the control and Rut-bpy group than in NSAIDs group (p<0.05).

**Conclusion:**

Rut-bpy may prevent renal histological changes in rats caused by meloxicam.

## Introduction

Non-steroidal anti-inflammatory drugs (NSAIDs) are commonly prescribed for the treatment of pain, inflammation and fever. On the other hand, NSAIDs can cause acute renal toxicity, which requires specialist review, renal biopsy, high-dose corticosteroid and/or immunosuppressant treatments, and will normally progress in chronic kidney disease (CKD)^1 , 2^ .

In this context, many studies demonstrated that nitric oxide (NO) modulation and its isoforms can change the renal diseases evolution^3 , 4^ . NO is soluble mediator produced by several cell types, including renal endothelial. The synthesis is catalyzed by NO synthase (NOS) with the presence of nicotinamide adenine dinucleotide phosphate (NADPH) and oxygen (O_2_)^5^ . In this reaction, one of the guanidine nitrogen atoms of L-arginine is oxidized resulting in N-hydroxy-L-arginine. Then, this product is transformed into L-citrulline and NO as a final product^5^ . Hence, the messenger-NO is physiologically synthesized in the kidneys, exerting vital functions in the blood flow homeostasis and renal excretion^3 , 6^ .

Currently, a metallopharmaceutical named Rut-bpy (Cis-[Ru(bpy)_2_(SO3)(NO)]PF_6_) can release NO *in vitro* and *in vivo* , as well as it can reduce inflammatory processes through NF-kB inhibition^7 , 8^ .

Considering the lack of studies associating NSAIDs with a NO-donor to prevent renal lesions, in this study we aimed to compare the prolonged use of NSAID alone and the use of NSAIDs together with new metallopharmaceutical NO donor. For this purpose, we studied: (1) the effects of meloxicam (a kind of NSAIDs) on renal histology in rat; (2) and the capacity of new metallapharmaceutical to attenuate meloxican-induced renal injury.

## Methods

All procedures and animal handling were conducted in accordance with the Guide for the Care and Use of Laboratory Animals from the Brazilian College of Animal Experimentation, after approval by the local ethics committee (protocol #62). The study was designed to minimize the number of animals required for the experiments.

Eighteen male Wistar rats weighing 200–230 g were used and maintained in the Laboratory of Experimental Surgery – LABCEX (UNICHRISTUS). Food and water were available *ad libitum* and the animals were maintained in the same environmental conditions in individual cages during a 12h/12h light/dark cycle.

### New metallopharmaceutical (Rut-bpy)

The Rut-bpy (cis-[Ru(bpy)2(SO3)(NO)]PF6) was synthesized and purified at the Department of Organic and Inorganic Chemistry, Universidade Federal do Ceará (Brazil), following procedures described elsewhere^9^ . Rut-bpy (working solution of 1.95 mM) was administered intraperitoneally at a dose of 0.15 mmol/kg (equivalent to 100 mg/kg). This dosage was based on previous experiments, which showed low toxicity to rats^10^ and benefits in others experimental model *in vivo* ^11^ .

### Experimental design

Animals were randomly divided into three groups of six animals each as follows:

- Control group (control): saline solution administration (vehicle) intraperitoneally injected for 10 days.
- NAIDs group (NSAID): meloxicam intramuscular administration (15 mg/kg), daily for 10 days.
- Rut-bpy group (NSAID+Ru): Rut-bpy administration (100 mg/kg), intraperitoneally injected associated with meloxicam (15 mg/kg - intramuscular) for 10 days.

### Histopathology

At the end of the experiments (tenth day) all animals were killed by an overdose of anesthetics (ketamine + xylazine). Renal tissue samples were collected from all animals. Tissue samples were fixed in formalin for 24 hours and transferred to 70% ethanol solution. Further processing included paraffin embedding and sectioning to generate 5-μm-thick tissue coronal sections to be mounted on glass slides. The slides were stained using hematoxylin and eosin and assessed by a pathologist (RP) following procedures described elsewhere^12^ .

### Analysis of fractal geometry (fractal dimension and lacunarity)

The fractal dimension (DF) and lacunarity (LAC) were calculated using box counting method^13 , 14^ with the aid of free image analyzer program IMAGE-J^®^ and the public domain FracLac plug-in (http://rsbweb.nih.gov/ij/).

During the preparation to avoid selection bias, in the present study it was chosen the option to analyze the entire histological renal image and for the analysis the TIFF image was transformed in binary automatically by the software, so it was transformed into an image consisting of black pixels (intensity 0) on white background (intensity 255).

The size of the box was programmed to increase progressively (box = 2, 3, 4, 8, 16, 32, 64). The data obtained were automatically organized in spreadsheets and electronic graphics by Image-J^®^.

Fractal dimension and lacunarity were evaluated using the automated block counting method, without interference from the researchers, and a binary image representing the object analyzed was divided into a series with several L-side squares. Next, the number N (L) of sheets containing the object (s) of interest were counted. The value of L varied exponentially, i.e., using L = 1,2,4,8 ..... to the limit of the image dimensions. The number that followed was calculated like this: once between the scale of the part of the images of the number of the plot and the digital number of images^15^ .

The DF corresponds to the alpha angular coefficient of the modulus-adjusted straight line and is defined as the slope of the straight line in the graph that correlates the degree of occupation of the space N (L) with the variable dimensional scales L to which it is analyzed. It reveals the level of regularity of an object between the different spatial scales (BOUDA M, 2016), being estimated by the radius of log change N (L) by the log change of scale L and having the following equation:

DF = variation log N (L) / variation log L

^15^ .

### Statistical analysis

Data were expressed as mean ± SD. After a Kolmogorov–Smirnov test of normality, parametric data were submitted to one-way analysis of variance followed by Tukey’s multiple comparison test. Histopathological data were analyzed with chi-square test. The level of statistical significance was set at 5%.

## Results

### Rut-bpy attenuated NSAID-induced histopathology, fractal dimension and lacunarity

**Figure 1:**
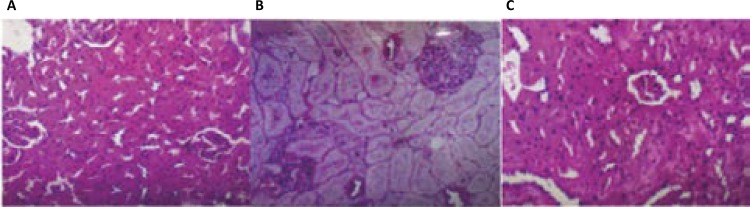
Representative images of the renal tissue histopathology of the Control, NSAID and NSAID+Ru groups. A) Normal slide of glomeruli and renal tubules of the Control group. B) Renal tissue of animals that received meloxicam, showed tubular congestion, thickening of membranes. C) Renal tissue of animals receiving meloxicam+Rut-bpy (group NASID+Ru) showed normal renal tubules and glomeruli. Image magnification x200. Abbreviations: NSAID=meloxicam; Ru = nitrosil ruthenium (Rut-bpy).

Histopathological examination of the renal tissue sections revealed that all rats (six animal - 100%) from meloxcam group (N=6) (NSAID) had necrosis, acute tubular congestion, vascular congestion, thickening of tubular membranes. In the group NSAID+Ru (N=6), only 1 animal (16.6%) presented a discrete eosinophilic infiltrate and mild vascular congestion; the other animals (five animals) had no histopathological alterations (Fig. 1). In the control group (N = 6) there was no animals with renal injury. The analysis of the discrepancy measurement between the observed and the expected frequencies (chi-square test) between the groups showed a significant difference between the groups NSAID vs. NSAID + Ru (chi-square = 14.49; p <0.05); there was no statistically significant difference between NSAID + Ru vs. Control (p> 0.05).

**Figure 2:**
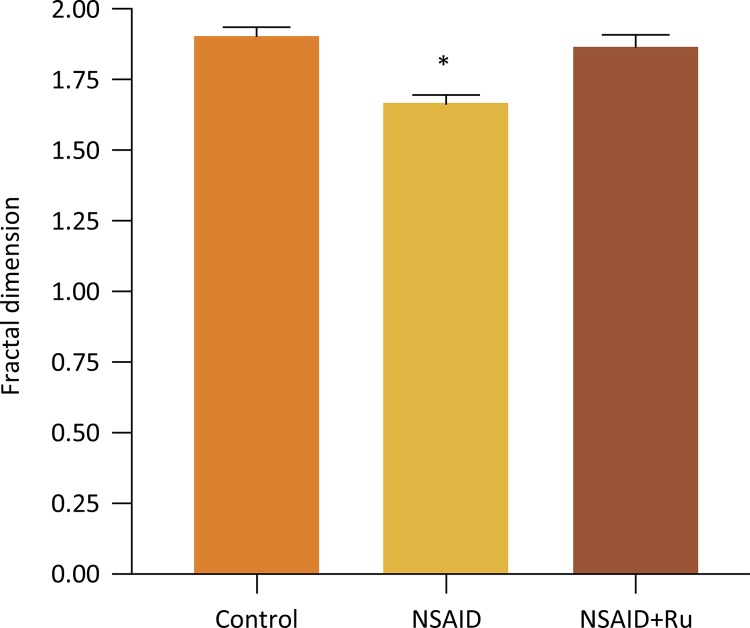
Rut-bpy administration associated with meloxicam. Fractal dimension analysis of the images of the renal tissue histopathology from six rats in each group. Values are expressed as mean ± SD. * *p* <0.05, as compared groups: NSAID *vs* . Control or NSAID *vs* . NSAID+Ru. Abbreviations: NSAID=meloxicam; Ru = nitrosil ruthenium (Rut-bpy).

The fractal dimension in the control and NSAID+Ru groups did not differ significantly ( *p* > 0.05). However, we found differences between control versus NSAID and NSAID versus NSAID+Ru, ( *p* <0.05). The Fractal dimension was greater in the control and NSAID+Ru group than in the group receiving NSAID alone (Fig. 2).

**Figure 3:**
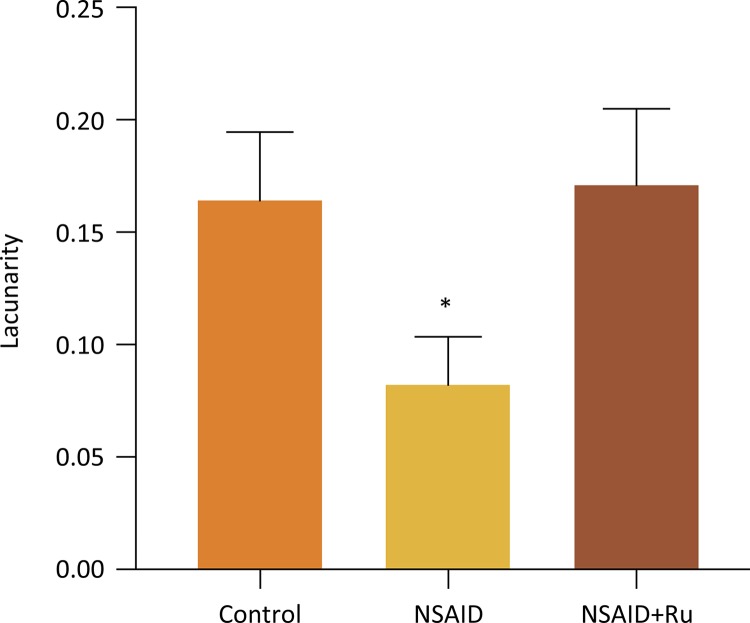
The lacunarity of the renal images from six rats in each group. Results are expressed as mean ± SD. * *p* <0.05, as compared groups: control vs. NSAID or NSAID *vs* . NSAID+Ru. Abbreviations: NSAID=meloxicam; Ru = nitrosil ruthenium (Rut-bpy).

The lacunarity was significantly smaller in the NSAID group as compared with the NSAID+Ru group ( *p* <0.05). The Control and NSAID+Ru groups did not differ significantly ( *p* >0.05) (Fig. 3).

## Discussion

Renal diseases can be associated with the use of several drugs, especially NSAIDs, leading to acute interstitial glomerulonephritis, which can become chronic and are also called papillary necrosis^16 , 17^ .

NSAIDs are one of the most prescribed drugs in the medical practice, especially to alleviate migraine, pain after surgery, arthritis, and dysmenorrhea due to their analgesic and anti-inflammatory effects in a wide range of clinical situations.

However, the use of NSAIDs should be restricted due to its adverse effects in the gastrointestinal tract and renal toxicity, which can be life-threatening due to acute renal toxicity.

Furthermore, nephrotic syndrome, a disorder caused by glomerulonephritis, diabetes, aminoglycosides, and indiscriminate use of NSAIDs, does not have an excellent therapeutic approach so far and its physiopathology lacks solid mechanisms.

The studies in vitro in renal tubular cell culture showed that immunoinflammatory disorder was associated to renal injury, with increased production of monocytes, MCP-1, endothelin-1, RANTES and NFkB^18 , 19^ .

In rats with proteinuria, inhibition of NFkB is able to protect them from nephritis^20 , 21^ . NFKB activated in podocytes. NFkB activation and NO production by NOSi are known to lead to cell damage, as shown in experimental rat model^8^ , and also promote glomerulonephritis^22^ .

NF-kB activation plays a dual role in cell death and survival, depending on cell type, developmental stage and apoptotic stimuli^23^ . The NO donors in experimental model have shown that they can inhibit NFkB activation and may prevent cellular damage caused by both activation of inflammatory factors and activation or inactivation of various protein kinases^24 - 26^ .

The NO donors, when applied in biological systems, release NO and are able to mimic the endogenous NO release response or replace an endogenous NO deficiency^27 , 28^ . Recently, many metallodrugs have been studied such as NO-donors or NO-scavengers, including ruthenium nitrosyl complexes^29^ .

New compounds obtained from ruthenium nitrosyl complexes and synthesized in the laboratory of the Department of Inorganic and Organic Chemistry, UFC (Cis-[Ru(bpy)2(SO3)(NO)]PF6, Ru-Bpy) according to the technique described by Silva *et. al.* ^9^ are water soluble and remain stable when exposed to the environment. These drugs can release NO and inhibit NFk-B *in vivo* ^8^ and *in vitro* ^7 , 30^ .

In this study, we used a new NO-donor metallopharmaceutic (Rut-bpy) simultaneously administered with meloxicam. This is the first report that associates a NO-donor and an NSAID with the potential to prevent pathologic modification of histopatologic and biochemistry in rat’s kidney.

In renal disease associated with the use of non-steroidal anti-inflammatory drugs (NSAIDs), histological changes often occur with predominantly lymphocyte infiltrate in the interstitium, and vacuolar degeneration of the proximal and distal tubules^17 , 31^ .

In this study, Wistar rats that received exclusively meloxicam showed renal histological changes (basal membrane thickening and tubulointerstitial nephritis), which are in agreement with other studies^32 - 34^ . On the other hand, rats that received meloxicam associated with nitrosyl ruthenium did not present such histological alterations and this is our main result. The histologic exam was performed by a blindly experienced pathologist.

These histological results support that the use of meloxicam associated with nitrosyl ruthenium may qualitatively reduce meloxicam-induced renal damage. Rut-bpy probably protects renal cells by nitric oxide (NO) donation at the vascular endothelium level, promoting renal protection when administrated in combination with meloxicam.

Other analyses that were performed with the histologic slides were: automatic dimension measures (fractal dimension and lacunarity) without human interference, which are able to determine details with the magnification of the image resolution and represent an estimate of the structural complexity of the tissue^35^ .

Fractal dimension (DF) is a quantifier of the geometric complexity of an object or image. It is able to do description and measurement of the self-similarity of the analyzed object, enables the comparison of parts of the structure with the structure as a whole^35 - 39^ . Fractal geometry has no units in the international system of unit, because it is a ratio between logarithms variation of the same unit.

Several studies have evaluated fractal dimensioning of histological slides to identify histological changes in several experiments, for example: assessing the degree of tumor invasiveness by quantifying the fractal dimension of the malignant epithelium/stroma interface^40^ , liver tissue with different degrees of cirrhosis due to hepatitis C^41^ , identification of dysplasia of lesions in oral cavity epithelium^42^ , in a study of quantification of the degree of myocardial cell rejection^43^ , estimation of nephron integrity^14^ and study pointed out that the vascular networks indirectly reflecting the architecture of stromal frameworks might have measurable and understandable differences between renal oncocytomas and ChRCCs (chromophobe renal cell carcinomas)^44^ .

Interestingly, the animal that received only meloxicam had a smaller fractal dimension than the control, probably due to nephritis caused by NSAID. This fact is congruent with the histological alterations found, since it had smaller fractal dimension and smaller similarity between others groups.

On the other hand, animals that received meloxicam and ruthenium simultaneously presented similar renal cytoarchitecture with the control group, corroborating the histological findings. In addition, we improved our result of fractal dimension evaluating other fractal parameter - the lacunarity.

The term lacunarity was introduced by Mandelbrot^39^ to describe features that can differentiate objects by varying the spatial distribution and the size of voids (gaps) that exist in a given object or image. Thus, a lacunarity is an element of fractal geometry that serves to describe the texture of an image^45^ .

A low lacunarity fractal object or image has been more homogeneous because it has, in probabilistic terms, the same size as the gaps, being the true inverse^46^ .

The tubular congestion, edema and thickening of membranes may reduce the gaps in the renal tissue, this spatial distribution of cytoarchitecture (gaps) may have been confirmed by the results of lacunarity which showed smaller in meloxican group than the other groups in study.

Fractal analysis, mainly through its fractal dimension and lacunarity parameters, has been increasingly used in the evaluation of images and inferences about the anatomical structure from the structural and architectural point of view^47^ .

This is the first time that renal tissues affected with nephrophatology by the use of meloxicam are under study with fractal dimension and lacunarity. Though there were many ways to measure the fractal dimension, we chose the ‘‘box-counting’’ method for both its simplicity and suitability of application for the objects seen in the histological sections.

## Conclusions

The metallodrug tested may play an important role in the prevention of NSAIDs-induced renal lesions. However, other studies are necessary to further investigate the ruthenium function and role in this pathology, including the evaluation of other renal markers. This drug may therefore be considered an attractive candidate for upcoming investigations with experimental renal disease by NSAID.

## Footnotes

1 Research performed at Laboratory of Experimental Surgery (LABCEX), Centro Universitário Christus (UNICHRISTUS), and Scientific Research and Teaching Institute (INPEC), Fortaleza-CE, Brazil. Part of Master degree thesis, Postgraduate Program in Minimally Invasive Technology and Health Simulation Area. Tutor: Prof. Marcio Wilker Soares Campelo.
